# The zebrafish model requires a standardized synthetic microbial community analogous to the oligo-mouse-microbiota (OMM12)

**DOI:** 10.3389/fmicb.2024.1407092

**Published:** 2024-06-05

**Authors:** Estefania Garibay-Valdez, Marcel Martínez-Porchas, Francisco Vargas-Albores, Diana Medina-Félix, Luis Rafael Martínez-Córdova

**Affiliations:** ^1^Centro de Investigación en Alimentación y Desarrollo, A.C. Biología de Organismos Acuáticos, Hermosillo, Sonora, Mexico; ^2^Departamento de Ecología, Universidad Estatal de Sonora, Hermosillo, Sonora, Mexico; ^3^Departamento de Investigaciones Científicas y Tecnológicas de la Universidad de Sonora, Universidad de Sonora, Hermosillo, Sonora, Mexico

**Keywords:** animal model, biological model, gut microbiota, syncoms, synthetic bacterial communities, synthetic microbiota

## 1 Introduction

The gut microbiota is a key regulator of various metabolic pathways in the host, including homeostasis, immunostasis, mucosal permeability, metabolic support, and even brain development. Animal models have substantially provided most of the current information about the gut microbiota, particularly gnotobiotic experimentation. In research, the term “gnotobiotic” describes a controlled environment where all microorganisms are known or excluded. This experimental setup allows for precise observation of interactions between host and their gut microbiota, giving a solid foundation for the study of the effects of specific microorganisms on host health and disease (Kubelkova et al., 2016).

Gnotobiotic models have been successfully applied to several animals, including mice, piglets, fish, insects, and nematodes. Mice have been the most used model for a variety of purposes, including predicting the human gut microbiota's response to a variety of factors in gnotobiotic mice (Faith et al., 2011), determining the effect of the gut microbiota on brain development (Lu et al., 2018), identifying gut microbe-host phenotype relationships (Faith et al., 2014), studying the role of probiotics and commensal microbiota in the development of the mucosal immune system, creating and characterizing communities of human gut microbes (Faith et al., 2010), and several others.

Depending on the study design, gnotobiotic models may consider germ-free, conventionalized (germ-free animals inoculated with the total fecal microbial community of their conventionally born and raised siblings), or standardized mice (germ-free mice inoculated with an *in vitro* standardized microbial consortia) (Turnbaugh et al., 2008; Williams, 2014). Standardized microbial consortia have been developed to permanently colonize gnotobiotic mice while providing fundamental functions to the host and serving as a comparative control. This is fundamental to researching and comprehending suspected interactions between the host and its microbiota and between varying components of such microbiota while representing a precise approach to translating associations into functions (Basic and Bleich, 2019). Fundamental aspects of gnotobiotic research are not only producing sterile embryos or larvae but sterilizing animal enclosures, food, and materials to avoid any microbial interference.

To advance research in this field, using standardized microbiota or synthetic bacterial communities (syncoms) is essential in studies involving gnotobiotic animals. The Oligo-Mouse-Microbiota (OMM12) is an example of a synthetic 12-bacterial species consortium created about a decade ago to facilitate functional microbiome research in mouse models (Brugiroux et al., 2016; Eberl et al., 2020). Despite other consortia developed for the same purposes (Stecher, 2021), the OMM12 is presently the most extensively employed strain collection for laboratory-based research. The bacterial strains have been fully sequenced and made publicly available; these strains were isolated from mice and are relatively easy to culture.

Conversely, synthetic bacterial communities like the OMM12 are not common or do not have the same standardization level as other animal models with valuable biological features. An example is the zebrafish (*Danio rerio*), the most used biological model. Here, we advocate for developing synthetic microbial communities or syncoms for the zebrafish model.

## 2 Oligo-mouse-microbiota 12

The OMM12 consortium offers several advantages over other defined consortia based on the available evidence. These benefits include a wide phylogenetic diversity, the accessibility of genomic sequences, strains from collections, and their stability for long periods in diverse mice strains (Brugiroux et al., 2016). Thus, OMM12 refers to a synthetic collection of microorganisms that mimic the naturally occurring microbiota in the mice's gastrointestinal tract that perform synergic functions for the host, including protection against pathogens. This consortium is formed by five phyla that include seven **Bacillota** species (*Clostridium innocuum, Clostridium clostridioforme, Lactobacillus reuteri, Enterococcus faecalis, Acutalibacter muris, Flavonifractor plautii, Blautia coccoides*), two **Bacteroidota** (*Bacteroides caecimuris, Muribaculum intestinale*), one **Actinomycetota** (*Bifidobacterium animalis* subsp. animalis), **Verrucomicrobia** (*Akkermansia muciniphila*), and **Pseudomonadota** (*Turicimonas muris*), all belonging to the mouse intestinal bacterial collection (miBC) (Lagkouvardos et al., 2016).

Research using OMM12 has focused on studying digestive tract colonization dynamics, infectious processes, mucosal immunology, microbial ecology, host-microbiome cross-talk, probiotics, etc (Brugiroux et al., 2016; Hernández-Mendoza et al., 2022). Beneficial adaptations have been developed for this bacterial consortia; for example, specific fluorescence *in situ* hybridization probes were designed and successfully proved to detect and quantify OMM12 (Brugiroux et al., 2022). Furthermore, considering that criticism has arisen due to the possible incompatibility of OMM12 with certain strains of mice, strains that are fully compatible with this bacterial consortium have been defined; in this case, C57BL/6 mice that have been stably colonized with OMM12 are referred to as stable defined moderately diverse microbiota mice (sDMDMm2) (Li et al., 2015). Recent evidence has demonstrated that the OMM12 inoculated germ-free mice reached the same stable gut microbiota composition regardless of the experimental facility (five European germ-free rodent facilities participated) (Eberl et al., 2020).

The OMM12 has been expanded with selected bacteria from a collection of over 200 bacterial strains. The collection was constructed using an enabled metagenome-educated prediction of synthetic communities to capture key functional differences between microbiomes. The OMM19 was elaborated by adding strains compensating for phenotype differences between OMM12 and specific pathogen-free mice (Afrizal et al., 2022). Thus, adaptations and improvements have been performed to address several research questions. However, other synthetic microbiota has been successfully used for decades, such is the case of the Altered Schaedler Flora (ASF) composed of only eight bacteria, a reduced consortium exempt of pathobionts, totally harmless to the intestinal cavity, and capable of being stable throughput generations and used in biomedical research. As a reduced consortium, advantages in applications, management and synthesizing have been reported (Wymore Brand et al., 2015).

## 3 Zebrafish

The unique natural features of zebrafish have made it the most used fish in biological research. Its ease of handling and manipulation at each stage of its life cycle, including reproduction, has led multiple laboratories to use it as an animal model, even above mice.

The fish undergoes external embryonic development (ovoviviparous) and produces abundant offspring that hatch within 48–72 h. The eggs have a translucent, semipermeable membrane that allows for easy observation of embryonic development (Castillo-Salas et al., 2022). Also, tissue regeneration of most organs, including vital organs like the heart and brain, is one of the particularities of the species and has been used to comprehend regeneration mechanisms in humans (Gemberling et al., 2013). Furthermore, this species displays early organogenesis, sharing similarities with mammalian organs. It boasts anatomical and genetic homologies with humans and possesses a well-developed immune system akin to humans—complete with innate and adaptive immunity; herein, around 70% of human genes are detected in zebrafish (Howe et al., 2013). As such, the fish species' immune mechanisms and receptors are preserved within the vertebrate class (Lieschke and Currie, 2007; Sullivan and Kim, 2008). The utilization of zebrafish as a model organism has proved to be an immensely valuable tool in progressing our understanding of various biological disciplines; for instance, it is useful in characterizing human diseases and identifying and testing new drugs to treat the diseases being modeled, developmental and toxicological research, etc. (Kari et al., 2007; Yang et al., 2009).

The similarities in the digestive system between zebrafish and humans have led to using this fish as a model to generate knowledge about the gut microbiota and related aspects requiring gnotobiotic specimens. Several techniques have been documented for producing zebrafish embryos, isolating and raising germ-free fish, and introducing microorganisms into the gut microbiota of zebrafish (Pham et al., 2008). In the zebrafish, for example, gnotobiotic models are carried out by producing germ-free fish and inoculating single bacterial species (monoassociation) such as *Aeromonas hydrophila, Pseudomonas aeruginosa, Escherichia coli* (Rawls et al., 2004, 2006). However, other methods involve euthanizing adult zebrafish, removing the intestinal contents, and transferring them to germ-free fish (Pham et al., 2008). However, standardizing a synthetic microbial community analogous to the OMM12 or similar is still a pending task for the zebrafish and any fish models.

Numerous studies have thoroughly documented the gut microbiota of zebrafish, revealing that it is influenced by various internal and external factors. Evidence indicates that the gut microbiota of juvenile and adult zebrafish is consistent across different habitats and stabilizes around the juvenile phase (~75 days) (Cornuault et al., 2022), with Proteobacteria and Fusobacteriota as the dominant phyla. In this regard, the most influential factor determining the gut microbiota of zebrafish is the internal condition established by the fish. Like in many other fish species, this microbiota maintains a symbiotic relationship with the host and plays crucial roles in protecting against pathogens, as well as in nutritional, endocrine, neural, and physiological functions (Vargas-Albores et al., 2023).

## 4 Discussion

**Figure 1:**
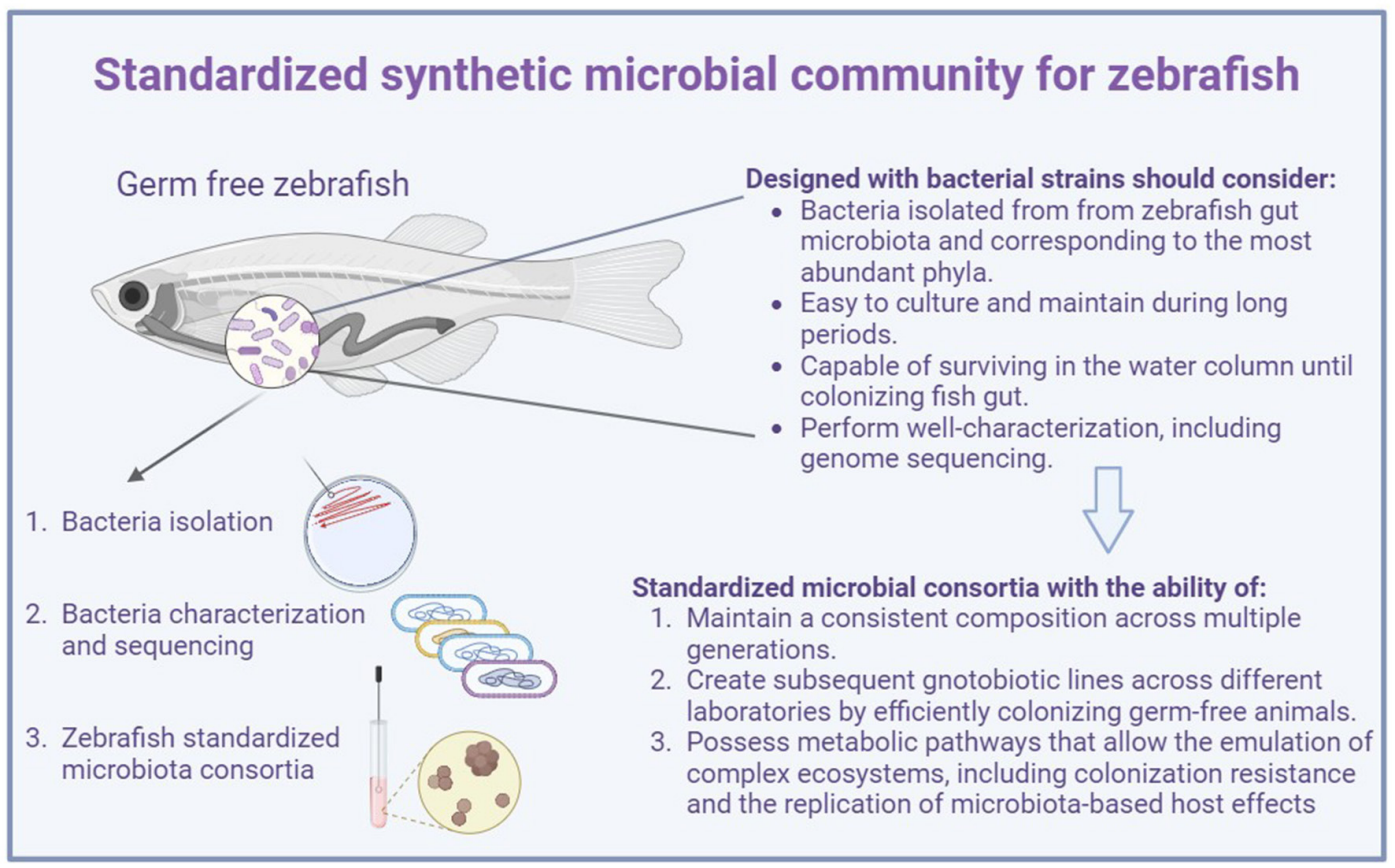
Considerations for designing a standardized synthetic microbial community for zebrafish.

Developing a synthetic microbial community to improve the zebrafish model focusing on studying the gut microbiota is still challenging. Designing and defining a synthetic microbiota for zebrafish may require not only considering bacterial strains belonging to the most abundant phyla but also coping with the criteria established for the OMM12, ASF, etc., which include three important characteristics that the microbial model system should possess: first, the ability to maintain a consistent composition across multiple generations reared under sterile conditions; second, perform efficient colonization in the germ-free animal to create subsequent gnotobiotic lines across different laboratories; third, possess metabolic pathways that allow for the emulation of complex ecosystems, including colonization resistance and the replication of microbiota-based host effects (Macpherson and McCoy, 2015) (Figure 1).

Approaches for developing a synthetic microbiota for the zebrafish have been made; for instance, 13 culturable and morphologically different bacterial species were reported as the most prevalent in zebrafish, including *Aeromonas hydrophila, Aeromonas sobria, Vibrio parahaemolyticus, Photobacterium damselae, Pseudomonas aeruginosa, Pseudomonas fluorescens, Pseudomonas luteola, Comamonas testosteroni, Ochrobactrum anthropi, Staphylococcus cohnii, Staphylococcus epidermidis, Staphylococcus capitis*, and *Staphylococcus warneri* (Cantas et al., 2012). Also, a meta-analytic approach performed in our laboratory, retrieving most of the available 16S sequences of the zebrafish gut microbiota reported in databases, revealed a zebrafish core microbiota that included representative bacteria from six phyla: Proteobacteria, Fusobacteriota, Planctomycetota, Firmicutes, Actinobacteriota, and Bacteroidota (Supplementary material 1). This information could serve as a first approach, but designing a synthetic microbiota for aquatic animals has additional challenges, starting with delivering viable microorganisms to the intestinal tract.

Gnotobiotic zebrafish models based on monoassociation provide a single bacteria through inoculation water at concentrations between 10^2^ and 10^4^ CFU/mL, trying to maintain such levels by microbial titration (Rawls et al., 2006). This technique may be applied to inoculate a bacterial consortium; however, this has complications associated with the biological requirements of the different bacteria, as all of them should be capable of at least surviving in water while incorporated in fish. Another strategy involves individual force-feeding; however, it is time-consuming, implies the manipulation and handling of animals, and is only viable for small groups of fish.

Developing a synthetic microbial community for fish and mice presents additional challenges due to the exclusive focus on cultivable bacteria. While the majority of microbes in the gut microbiota are prokaryotic, the eukaryotic counterpart also plays a significant biological role. Critics of synthetic microbiota based solely on bacteria argue that although designed consortia can confer benefits and restore functions in gnotobiotic models, they do not fully replicate the multitrophic reality (Vargas-Albores et al., 2023). Besides, such synthetic microbial communities only contain cultivable bacteria, which some environmental microbiologists estimate is a minor proportion compared to non-culturable bacteria, while others argue that such a percentage is significantly higher in bacterial communities associated with an animal host (Wade, 2002; Steen et al., 2019); however, this is a limitation that cannot be easily overcome due to the difficulty of developing culture media with conditions that allow the growth of a higher percentage of bacteria.

Although there have been difficulties, utilizing synthetic microbial communities like the OMM12, ASF, and others in mice has yielded valuable insights into the gut microbiota and represents progress toward understanding the intricate interplay between the microbiota and its host. Consequently, developing and standardizing a synthetic microbial community for zebrafish would undoubtedly bolster the production of fundamental and applied scientific knowledge while taking advantage of its high fecundity, external fertilization, optical transparency, and rapid development. Furthermore, the relationship between gut microbiota and major neuromodulator systems, including neurotransmitter receptors, transporters, and enzymes involved in synthesis and metabolism, could be studied, as they are similar to those observed in humans and rodents.

## Author contributions

EG-V: Conceptualization, Supervision, Writing – original draft, Writing – review & editing. MM-P: Conceptualization, Supervision, Visualization, Writing – original draft, Writing – review & editing. FV-A: Conceptualization, Supervision, Writing – original draft, Writing – review & editing. DM-F: Resources, Visualization, Writing – review & editing, Writing – original draft. LM-C: Visualization, Writing – review & editing, Writing – original draft.
