# Supplementary material for: The zebrafish model requires a standardized synthetic microbial community analogous to the oligo-mouse-microbiota (OMM12)
Source: Front Microbiol. 2024 Jun 5;15:1407092. doi: 10.3389/fmicb.2024.1407092 (PMC11188439; doi:10.3389/fmicb.2024.1407092)
Supplement: Supplementary file 1 [file Table_1.DOCX]

Supplementary Material

# Supplementary Material 1. Bioprojects datasets of gut microbiota analysis with Zebrafish (*Danio rerio*) used in this meta-analysis.

| **Bioproject** | **16S ARNr region** | **Sequencing platform** | **Sample size** | **Life stage** | **Survival** | **Diet** | **Reference** |
| --- | --- | --- | --- | --- | --- | --- | --- |
| PRJNA378677 | V4 | Illumina HiSeq | 62 | 21 days dpf (larvae) (Juvenile reported) | 90 -100% | WT AB/Tübingen fish, fed with live brine shrimp. | Burns *et al.,* (2017) |
| PRJNA791519 | V3-V4 | Illumina MiSeq | 5 | 1 month old reporthed - Larvae or Juvenile | 100% | The control-check diet (CK) was supplemented with soybean oil at 60 g/kg | Ding *et al.,* (2022) |
| PRJNA601771 | V3-V4 | Illumina MiSeq | 4 | Adult | 100% | Fish were fed twice per day ad libitum with a commercial fish diet (Wardley®) and maintained at a photoperiod of 14:10 h of light: dark | González-Penagos *et al.,* (2020) |
| PRJNA628820 | V3-V4 | Illumina HiSeq | 3 | Adult | Not reported | Control group (0.01% DMSO) | Jiang *et al.,* (2020) |
| PRJNA528701 | V3-V4 | Illumina MiSeq | 3 | Larvae | Not reported | was maintained at a 12:12 h light/dark photoperiod and was used as control (CT) | Lutfi *et al.,* (2021) |
| PRJNA699145 | V3-V4 | Illumina MiSeq | 10 | Adult | Not reported | Fed with Artemia salina | Orso *et al.,* (2021) |
| PRJNA899980 | V3-V4 | Illumina Miseq | 8 | Adult (3 months) | Not reported | The zebrafish were fed twice daily at 09:00 a.m. and 17:00 p.m. with a commercial diet (Tetra Bits Complete) | Liu *et al.,* (2023) |
| PRJNA728442 | V3-V4 | Illumina | 4 | Adult (6-8 months) | 100% | Fish injected with PBS buffer were used as controls | Pacheco *et al.,* (2021) |
| PRJNA565801 | V4-V5 | Illumina HiSeq | 189 | Larvea to adult | Not reported | Zebrafish were fed with cultured Paramecium (5–8 dph), 20 µm mesh filtrated boiled egg yolk (9–11 dph), live brine shrimp (12–19 dph), and a standard dry fish food from 20 dph onward. | Xiao *et al.,* (2021) |
| Note: zebrafish developmental stage substantially explains the gut microbial community succession |  |  |  |  |  |  |  |
| PRJNA751025 | V3-V4 | Illumina MiSeq | 9 | Embryos to adult | Not reported | fed with commercial non-medicated feed (AQUAFIN, Malaysia; 4% of fish body weight) twice per day. | Xue *et al.,* (2023) |
| PRJNA821478 | V3-V4 | Illumina NovaSeq | 3 | Adult | 100% | The fish were fed three times/day with a mixture of hatched Artemia nauplii and commercial food (Bonuses). | Yuan *et al.,* (2023) |
| PRJNA543612 | V3-V4 | Illumina MiSeq | 23 | Adult | Not reported | Normal feed and regular filtered water. | Zhang *et al.,* (2021) |

**
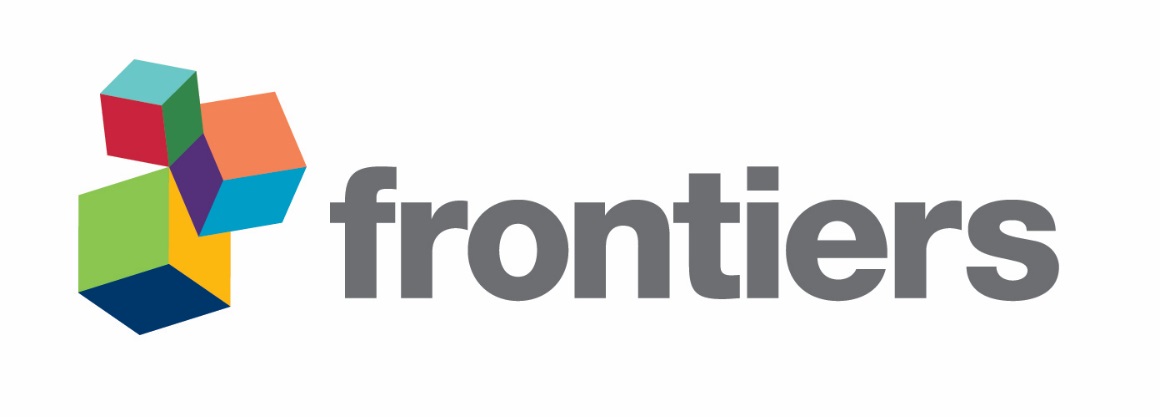
**
